# Evaluation of a pilot healthy eating intervention in restaurants and food stores of a rural community: a randomized community trial

**DOI:** 10.1186/s12889-015-1469-z

**Published:** 2015-02-12

**Authors:** Ana P Martínez-Donate, Ann Josie Riggall, Amy M Meinen, Kristen Malecki, Anne L Escaron, Bev Hall, Anne Menzies, Gary Garske, F Javier Nieto, Susan Nitzke

**Affiliations:** Department of Population Health Sciences, University of Wisconsin, Madison, WI USA; Obesity Prevention Network, University of Wisconsin, Madison, WI USA; University of California, Los Angeles, USA; Waupaca County NuAct Coalition, Waupaca, WI USA; Portage County Activity and Nutrition Coalition, Stevens Point, WI USA; Department of Nutritional Sciences, University of Wisconsin, Madison, WI USA

**Keywords:** Healthy eating, Food environment, Restaurant interventions, Food store interventions, Rural communities

## Abstract

**Background:**

Research suggests that the food environment influences individual eating practices. To date, little is known about effective interventions to improve the food environment of restaurants and food stores and promote healthy eating in rural communities. We tested “Waupaca Eating Smart ” (WES), a pilot intervention to improve the food environment and promote healthy eating in restaurants and supermarkets of a rural community. WES focused on labeling, promoting, and increasing the availability of healthy foods.

**Methods:**

We conducted a randomized community trial, with two Midwestern U.S. communities randomly assigned to serve as intervention or control site. We collected process and outcome data using baseline and posttest owner and customer surveys and direct observation methods. The RE-AIM framework was used to guide the evaluation and organize the results.

**Results:**

Seven of nine restaurants and two of three food stores invited to participate in WES adopted the intervention. On a 0-4 scale, the average level of satisfaction with WES was 3.14 (SD=0.69) for restaurant managers and 3 (SD=0.0) for store managers. On average, 6.3 (SD=1.1) out of 10 possible intervention activities were implemented in restaurants and 9.0 (SD=0.0) out of 12 possible activities were implemented in food stores. One month after the end of the pilot implementation period, 5.4 (SD=1.6) and 7.5 (SD=0.7) activities were still in place at restaurants and food stores, respectively. The intervention reached 60% of customers in participating food outlets. Restaurant food environment scores improved from 13.4 to 24.1 (p < 0.01) in the intervention community and did not change significantly in the control community. Food environment scores decreased slightly in both communities. No or minimal changes in customer behaviors were observed after a 10-month implementation period.

**Conclusion:**

The intervention achieved high levels of reach, adoption, implementation, and maintenance, suggesting the feasibility and acceptability of restaurant-and food store-based interventions in rural communities. Pilot outcome data indicated very modest levels of effectiveness, but additional research adequately powered to test the impact of this intervention on food environment scores and customer behaviors needs to be conducted in order to identify its potential to promote healthy eating in rural community settings.

**Electronic supplementary material:**

The online version of this article (doi:10.1186/s12889-015-1469-z) contains supplementary material, which is available to authorized users.

## Background

Obesity is a complex public health problem with serious health and economic consequences [1,2]. In the US, over two thirds of adults are overweight or obese [3]. The problem is even more acute in rural areas where obesity rates tend to be higher [4] and resources for obesity prevention efforts are typically scarcer [5]. Research indicates that a healthy diet plays a critical role in preventing obesity and chronic diseases, however almost all Americans fail to adhere to dietary recommendations [6].

Growing evidence indicates that the nutrition environment, encompassing food access, availability, pricing, quality, and promotion, [7] is linked to individual dietary behaviors [8,9] and obesity rates [10]. Restaurants and food stores are two main domains of the community nutrition environment [7] and represent suitable settings for interventions aimed to promote healthier dietary practices and curb the obesity epidemic at a population level.

Previous research on community-based interventions promoting healthy eating in restaurants and stores has provided some evidence of feasibility, but data on their effectiveness to positively alter the nutrition environment and patrons’ purchasing behaviors is still inconclusive [11-13]. While evidence supporting these interventions is growing, gaps remain in the literature. For instance, although 19% of Americans live in rural areas [14] and 40% of rural adults are obese, [4] few studies have evaluated restaurant or store interventions in rural or semi-rural areas [15]. Additionally, almost all interventions have focused exclusively on either restaurants or food stores, missing the opportunity for synergy between these two domains. Furthermore, there is a need for stronger and more comprehensive evaluation designs using baseline measures, comparison groups, and data beyond efficacy or effectiveness to better assess the public health impact of these interventions [13,16].

The purpose of this study is to pilot test a community-level intervention to improve the nutrition environment and promote healthy eating in restaurants and food stores of a rural community. The evaluation emphasized acceptability and feasibility and was guided by the RE-AIM framework, [17] a model that considers the public health impact of an intervention as determined not only by its effectiveness or efficacy, but also by the ability to reach the intended audience (Reach), the likelihood of being adopted by the intended organizations or settings (Adoption), the extent and fidelity of implementation (Implementation), and the likelihood of being sustained over time (Maintenance). By attending to these multiple dimensions for an intervention designed to meet the needs of a rural community, our study was designed to inform a future effectiveness trial aimed to expand the evidence base related to community-based approaches to promote healthy eating and curb the obesity epidemic in rural U.S. communities.

## Methods

### Study design

A pilot randomized community trial was conducted in two Midwestern rural communities randomly assigned to serve as either an intervention or a control site. The two communities were located about thirty miles apart in adjacent counties. They were selected based on their relatively small population size (6,000 to 26,000 residents), similar socio-demographic profile, presence of an active Nutrition and Physical Activity (NPA) coalition, and absence of other major healthy eating initiatives prior to the beginning of the study. Seven restaurants and two food stores agreed to participate in the intervention community; likewise seven restaurants and two food stores matched in approximate customer volume were recruited to serve as comparison sites in the control community. Multi-modal methods were used for evaluation of the intervention on the RE-AIM dimensions (Table 1). All study procedures were approved by the Institutional Review Board at the University of Wisconsin-Madison.

**Table 1:** **Evaluation of WES along RE-AIM dimensions**

**RE-AIM dimension**	**Definition**	**Data source**	**Evaluation**
Reach	Percent and characteristics of individuals reached by an intervention	Customer surveys	Descriptive statistics of post-test customer awareness, logo recognition, and assessment of materials (understandable, appealing, helpful)
Effectiveness			
• Environment (nutrition environment)	Changes in nutrition environment scores attributable to intervention	NEMS data	Paired t-tests of pre/post nutritional environment scores stratified by community
• Individual (customer and owner behaviors and theoretical mediators)	Changes in customer behaviors and attitudes attributable to intervention	Customer surveys 1-month and 10-months after the intervention	Adjusted multiple regression models of customer satisfaction and choices
Adoption	Percent and characteristics of outlets of settings that agreed to participate	Program records	Descriptive statistics of managers approached that agreed to participate in the intervention
Implementation	Extent to which intervention is implemented as planned	Direct observation after 5-month intervention period	Descriptive analysis of strategy implementation by food outlet and by strategy
Maintenance	Extent to which intervention is likely to be sustained over time	Direct observation after 10-month intervention period	Descriptive analysis of strategy implementation
		Owner surveys	Descriptive analyses of manager interest in continued participation at post-test

### Waupaca Eating Smart

Waupaca Eating Smart (WES) was a pilot intervention developed by a university-based academic team and two local nutrition and physical activity (NPA) coalitions. WES was informed by a formative assessment and guided by a social ecological model (SEM). The SEM posits that health behaviors are the result of the interplay between individual attributes (i.e. knowledge, attitudes, etc.) and environmental factors, with emphasis on the role of social, policy, and built environment factors [18]. We used a participatory research approach, [19] characterized by equitable collaboration between community members and researchers in all aspects of the research process [20]. WES also incorporated social marketing methods [21]. Social marketing is the application of commercial marketing techniques to positively influence health behaviors, using a consumer-oriented process and targeting changes in social norms as a mechanism to change behaviors [22]. WES was implemented simultaneously in 9 food retail establishments (i.e. 7 restaurants and 2 supermarkets) from October 2011 through July 2012. Core WES strategies included increasing availability, point-of-purchase/labeling, and promotion of healthier items in local restaurants and grocery stores. Specific intervention activities, such as displaying a window cling or using menu inserts, were implemented for each of these broad strategies (See Table 2 for a complete list of WES strategies). To formally participate in WES, restaurant and store managers signed a written agreement to implement a minimum of 3 intervention activities and participate in the evaluation of WES.

**Table 2:** **Selection, implementation, and maintenance of WES strategies across outlets and time points: direct observation data**

**Possible restaurant strategies**	**Restaurants selecting and implementing**			**Possible store strategies**	**Stores selecting and implementing**		
	**Initially selected** ^**a**^	**Observed at mid** ^**b**^	**Observed at post** ^**c**^		**Initially selected** ^**a**^	**Observed at mid** ^**b**^	**Observed at post** ^**c**^
Window cling	7	7	7	Window cling	2	2	2
Signs on the counter	2	4	3	Signs on service counter	2	2	2
Signs by the register	2	3	0	Signs by registers	2	2	2
Menu stickers	3	1	2	Staff wears WES pins	0	0	0
Menu inserts	6	3	2	Signs for local produce	1	0	0
Table tents	4	5	5	Point-of-purchase signs	2	2	2
Promotion of WES items by wait staff	7	0	0	Healthy recipes	2	2	2
Wait staff can explain WES*	7	7	6	Shopping list for healthy recipes	0	0	0
Wait staff can recommend WES items*	7	7	6	In-store display**	2	2	2
Restaurant offers one or more WES meal	7	7	7	Bag stuffers**	2	2	0
				Staff can explain WES*	2	2	2
				Staff can recommend WES items*	2	2	1
	**Strategies in restaurants (Out of 10 possible strategies)**				**Strategies in stores (Out of 12 possible strategies)**		
	**Initially selected** ^**d**^	**Mid** ^**e**^	**Post** ^**f**^		**Initially selected** ^**d**^	**Mid** ^**e**^	**Post** ^**f**^
**Total Number of Strategies, Mean (SD)**	7.73 (1.29)	6.29 (1.11)	5.43 (1.62)		9.5 (0.5)	9.0 (0.0)	7.5 (0.7)

^a^Number of outlets selecting each strategy prior to launching the intervention.

^b^Number of outlets implementing the strategy at mid-intervention point (5 months after launching the intervention).

^b^Number of outlets implementing the strategy at post-intervention point (10 months after launching the intervention).

^d^Average number of intervention strategies per outlet selected prior to launching the intervention.

^e^Average number of intervention strategies per outlet implemented at mid-intervention point (5 months after launching the intervention).

^f^Average number of intervention strategies per outlet implemented at post-intervention point (10 months after launching the intervention).

*Assessed upon probing by WES staff during audit.

**Assessed based on observation by WES staff and/or reports from outlet managers.

Nutrition criteria were developed by reviewing those included in other healthy eating interventions [23,24] and considering what may be feasible and sustainable in the intervention community. “WES-approved” bundled meals (entrée and side dish) contained less than 700 calories and included a cup of fruit or vegetables (excluding French fries); “WES-approved” side dishes contained less than 300 calories and included a half-cup of fruit or vegetables.

A local registered dietitian in the NPA coalition analyzed menu items at individual restaurants to identify or create one to three “WES-approved” meals at each restaurant. Promotional and point-of-purchase materials including window clings, table tents, menu stickers, and menu inserts were developed by the academic team and NPA coalition to identify and promote these meals and other healthy eating practices. At food stores, bi-monthly displays staffed by the NPA coalition offered samples of WES side dishes prepared by the store; WES materials included signs for produce, recipes for bundled meals, and fliers with healthy eating tips.

Additional promotional activities were implemented at the community level to influence social norms regarding healthy eating. The NPA coalition selected a local, well-known, member of the community to help promote WES. This local “celebrity” promoted WES by eating at each WES restaurant twice over the evaluation period and writing a total of 14 articles in the local newspaper about his experience. An additional six newspaper articles published in local media featured WES. A blog and Facebook page promoted WES by circulating articles, healthy eating tips, recipes, and information about WES. Additional details on WES development process and content are presented elsewhere [25].

### Evaluation procedures and measures

#### Customer surveys

For evaluation of customer-level reach and effectiveness, we conducted interviewer-administered pre-and posttest intercept surveys with convenience samples of customers in the intervention and comparison outlets. Approximately thirty customer surveys per outlet were completed during a two-week period 1-month before and 10-months after implementation of the intervention. A minimum of two survey shifts were purposely selected to cover an equal number of lunch and dinner times for restaurants (morning, midday, and evening times for stores), as well as weekdays and weekend days, in each food outlet. During each selected survey shift, each customer exiting the food outlet was approached consecutively, screened for eligibility (i.e. 18 years or older, fluent in English, and having just eaten or purchased food in the restaurant or store), and invited to complete a paper-based self-administered anonymous survey. Verbal consent was obtained from survey participants. The survey took an average of 6-7 minutes to complete. Customers who completed the survey received a $2 gift certificate for the food outlet.

Pretest and post-test intercept customer surveys included questions on satisfaction with healthy options available, perceived healthiness of the foods purchased, and whether they purchased any foods promoted as healthy in the outlet. At post-test in the intervention community only, questions also asked about WES name and logo recognition, exposure to WES activities/materials/messages, and, among those exposed, degree of appeal, ease of interpretation, helpfulness to decide purchase, and whether the respondent ordered/purchased any WES foods. Answers included five-point Likert scales (e.g., 0=not a lot to 4=a great deal) or Yes/No choices.

### Nutrition environment measures

To evaluate the impact of WES on the food environment we used the Nutritional Environment Measures Surveys (NEMS). The NEMS has been previously validated, showing high degrees of inter-rater and test-retest reliability, and good validity properties [26,27]. One month before and 10 months after launching of WES, a trained research assistant assessed the food environment in participating outlets in the intervention and comparison communities using NEMS. The rater was external to the research team and not informed about the WES or the purpose of the environment audit prior to conducting the assessments. NEMS is an observational audit tool of nutrition environments. Restaurants received points for a variety of measures related to availability of healthier foods, pricing, nutrition information or healthy symbols, and signage facilitating healthy eating (up to 90 points). Stores received points for availability, pricing, and quality of healthier foods (up to 66 points). For both, restaurant and stores, higher overall scores represent conditions more conducive to healthy eating [26,27].

### Manager surveys

For evaluation of implementation and maintenance, post-intervention interviewer-administered surveys were conducted with owners, operators, or managers (hereafter referred to as “managers”) of participating outlets at the intervention community (n=9). Written consent was obtained prior to enrollment. No incentives were offered to managers for completing the surveys. Post-intervention manager survey questions included intentions to continue implementing WES (from 0=not likely to 4=very likely), perceived impact on their business (0=very negative to 4 very positive), and overall satisfaction with WES (0=not at all to 4=a great deal).

### Direct observation of WES implementation

Coalition members completed unannounced visits to WES outlets to conduct direct observation of WES activity implementation at mid-intervention point (i.e. 5 months after launching of WES) and at posttest (i.e. 10 months after the intervention). Using a checklist, coalition members noted whether the activities were in place or ongoing at the time of the visit (possible activities are listed in Table 2). Based on these observations, each of the possible strategies was coded as 1=implemented or 0=not implemented on the participating outlets by mid-intervention and post-intervention point.

### Analysis

Descriptive statistics were estimated to evaluate reach, adoption, implementation, and maintenance. To measure intervention effects on restaurant nutrition environments, paired t-tests were conducted separately for each community. Given the small number of supermarkets (N=4), descriptive statistics were used in place of formal statistical testing to examine changes in store nutrition environments.

This pilot study was not powered to detect significant differences in outcomes, but to test the feasibility and acceptability of WES and inform a future effectiveness study. However, we explored intervention effects on customer satisfaction, perceptions, and behaviors, using multiple linear and logistic regression models. The main predictors were the community (intervention versus comparison), time (pretest versus post-test), and intervention effect (interaction term community*time). Regression models accounted for the clustering of the data within outlets and were adjusted for age, gender, education, whether the participant was a local community resident or a visitor, and day of the survey (weekend versus weekday). For restaurants, models were further adjusted for time of day (breakfast/lunch versus dinner time) and whether participants were celebrating a special event or holiday (yes versus no) at their most recent meal in the restaurant. All analyses were conducted with Stata/SE 12.0 for Mac (StataCorp, LP, College Station, TX).

## Results

### Adoption

Seven (78%) out of nine restaurants and two (67%) out of three food stores approached for participation signed written agreements to participate in WES. Of the restaurants, six were locally owned single-unit restaurants and one was a franchise chain restaurant. Participating restaurants represented a range of outlets including one fast-casual restaurant and six sit-down restaurants, which included three American style restaurants, two cafés, and one pizza restaurant. The two restaurants that declined participation were American style sit down restaurants. The two food stores that participated were both larger chain grocery stores. One was a corporate store and the other was a franchise. The food store declining participation was a convenience store. Participating outlets represented a third of all restaurants and the two largest food stores in the intervention community.

### Implementation & maintenance

#### WES activities observed

None of the WES activities were found to be in place in restaurants or stores prior to the intervention. After launching of WES, all restaurants successfully implemented several broad strategies, including offering healthy bundled meals, training wait staff to promote the program, and displaying promotional materials around the restaurant. Out of a possible 10 WES activities offered to the outlets, on average restaurants selected 7.42 (range 7-9) strategies prior to the beginning of the intervention, had implemented 6.3 (range 5-8) activities by mid intervention point, and, of these, 5.4 (range 2-7) activities remained in place one month post-intervention (Table 2).

The two supermarkets also implemented a number of activities, including offering recipes and shopping lists for healthy bundled meals, offering in-store displays with healthy samples, placing promotional materials around the store, providing bag stuffers with healthy tips, and posting point-of-purchase signs for fruits and vegetables (Table 2). Out of a possible 12 activities, stores selected 9.5 (range 9-10) strategies prior to launching of WES, had implemented 9 activities by mid intervention point (9 at both stores) and, of these, 7.5 activities remained in place one month post-intervention (range 7-8).

#### Manager intention to sustain WES

Managers were 42 years old on average (SD=7.32). Five (56%) were males and four were females (44%). About 44% had completed at least some college studies. Five (33%) were owners and four (44%) were managers. On a 0-4 scale (0=not likely, 4=very likely), the average likelihood of continuing implementing WES at the end of the 10-month evaluation period was 2.86 (SD=0.90) for restaurant managers and 3.5 for store managers (SD=0.71). On a 0 (very negative) to 4 (very positive) scale, the average impact on business was 3.0 (SD=0.0) and 2.0 (SD=0.0) for restaurant and store managers, respectively. The average level of satisfaction with WES was 3.14 for restaurant managers (SD=0.69) and 3 (SD=0.0) for store managers, on a 0 to 4 scale (0=not at all, 4=a great deal).

### Reach

We obtained a response rate of 50.3% and 38.5% for customer surveys in restaurants and stores, respectively. Samples sizes, demographic, and contextual characteristics of survey respondents are shown in Table 3.

**Table 3:** **WES customer intercept surveys: demographic and contextual characteristics**

	**Restaurant customers**				**Store customers**			
	**Intervention community**		**Comparison community**		**Intervention community**		**Comparison community**	
**Variable**	**Pre (n=168)**	**Post (n=151)**	**Pre (n=215)**	**Post (n=187)**	**Pre (n=96)**	**Post (n=203)**	**Pre (n=99)**	**Post (n=203)**
Gender (female), %	60.8	64.2	61.0	67.2	75.8	73.9	72.2	68.7
Age, Mean (SD)	55.0 (14.8)	56.2 (18.1)	50.6 (18.3)	52.3 (18.7)	56.7 (15.1)	58.9 (15.2)	54.0 (16.9)	55.1 (17.5)
Education (completed college degree), %	44.6	39.1	46.0	53.0	29.5	41.0	34.0	40.7
Local resident,^a^ %	47.6	48.3	63.0	58.3	76.8	65.2	82.7	77.5
Survey time (lunch/morning or midday)^b^, %	46.4	55.6	47.4	51.9	56.3	66.9	66.7	70.0
Survey day (weekday),^c^ %	32.7	60.3	38.6	58.8	37.5	37.9	41.4	40.6
Celebrating special occasion (yes),^d^ %	19.6	18.0	15.7	14.0	na	na	na	na

^a^Percentage of participants who were residents of the community where they were surveyed.

^b^Percentage of participants who were surveyed at lunch time (versus dinner) in restaurants or in the morning or midday (versus afternoon or evening) in stores.

^c^Percentage of participants who were surveyed on a weekday versus the weekend. For restaurants, Friday evening. Saturday, and Sunday were considered the weekend; for stores, all Friday-Sunday was considered the weekend.

^d^Percentage of restaurant participants who reported they were celebrating a special occasion during their most recent meal at the restaurant.

At post-test in the intervention community, 51.0% of exiting restaurant customers (N=151) reported they had heard of WES, 60.9% recognized the WES logo, and 36.9% had noticed the WES logo in the restaurant they just exited. On a 0-4 scale (0: not at all, 4: a great deal), restaurant customers who noticed WES materials reported that, on average, ease of understanding WES materials was 3.1 (SD=0.9), level of appeal was 2.1 (SD=1.3), and helpfulness in deciding what to order was 0.9 (SD=1.3). Post-test customer survey results show 12.1% of patrons surveyed in participating restaurants reported having ordered a WES approved meal. These customers represented 48.8% of customers who noticed WES signage in a participating restaurant.

In stores, 50.5% of customers (N=201) had heard of WES in the intervention community at post-test, 59.1% recognized the WES logo, and 50% had noticed the WES logo in the store they just visited. Of store customers who noticed WES in the store, 37.8% reported seeing deli samples of WES side dishes. On average, store customers who noticed WES materials reported that ease of understanding WES materials was 3.0 (SD=0.9), level of appeal was 2.3 (SD=1.1), and helpfulness in deciding what to order was 1.2 (SD=1.2) on a 0-4 scale (0: not at all, 4: a great deal). About 15.5% of surveyed customers reported purchasing foods promoted by WES signs or materials. These customers represented 68.9% of store customers who noticed WES signage in the store.

### Effectiveness

#### Nutrition environment

**Figure 1:**
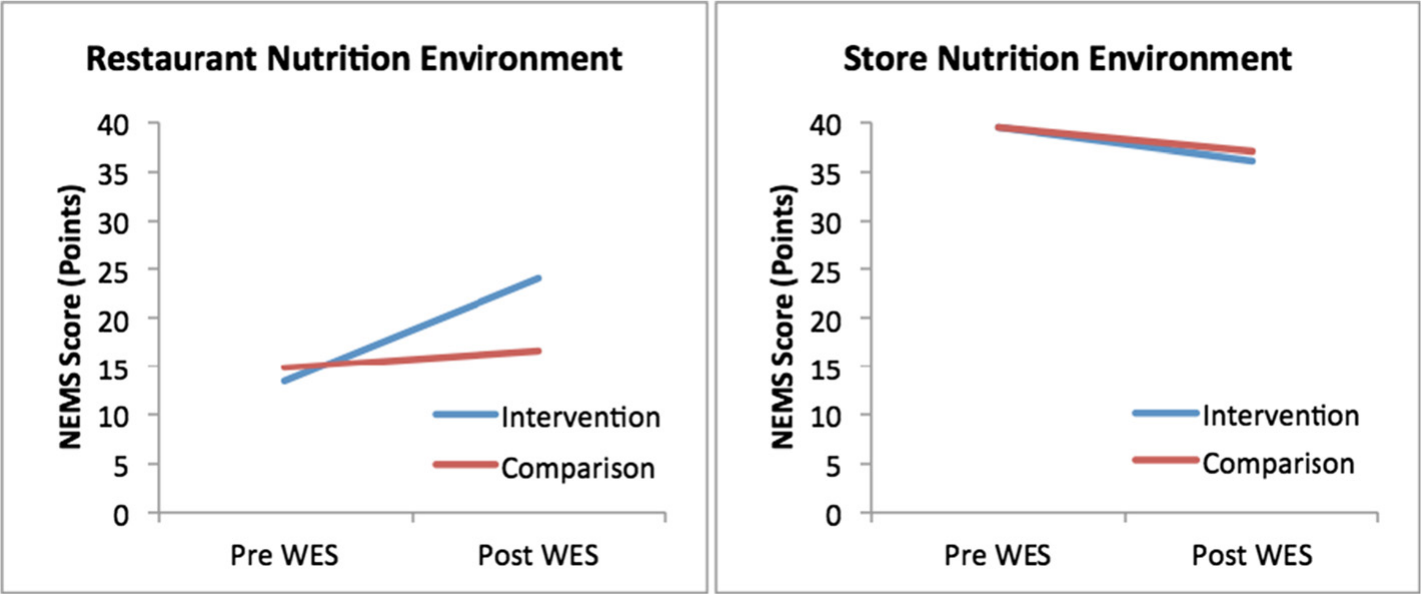
**Nutrition environment scores before and after “Waupaca Eating Smart”.** Detailed Legend. Food environment scores measured with the Nutrition Environment Measurement Survey for Restaurants (NEMS-R) and for Stores (NEMS-S) in food outlets located in the intervention and comparison community (7 restaurants and 2 supermarkets per community) before and after implementation of WES. Higher scores reflect a food environment more conducive to healthy eating. Pre WES reflects nutrition environment scores 1 month prior to implementing the intervention. Post WES reflects nutrition environment scores 10 months post intervention.

In restaurants, assessment of the nutrition environment showed that the average NEMS score for the 7 restaurants in the intervention community increased from 13.4 to 24.1 (t=3.74, p=0.010) after the implementation of WES (Figure 1). In the comparison community, scores remained similar before and after the intervention (14.9 to 16.6; t=0.560, p=0.596).

NEMS scores in food stores decreased slightly in both communities after the intervention period, from 39.5 to 36.0 in the intervention community and from 39.5 to 37.0 in the comparison community (Figure 1).

### Consumers

Results from regression models suggest improvements in satisfaction with fruit and vegetable choices and satisfaction with low-calorie choices in restaurants in both communities, with no evidence of greater improvements in the intervention community compared to the comparison community (Table 4). A trend suggesting an increase in the percentage of customers who reported having ordered any food items promoted as healthy in the intervention community compared to the comparison community was observed. However, the effect did not reach statistical significance (AOR=2.23, p=.094). No effects on the perceived degree of healthiness of the last meal consumed in the restaurant were observed.

**Table 4:** **Customer attitudes and purchase survey data before and after implementing “Waupaca Eating Smart”**

**Variable**	**Community**	**Time**		**Community effect(Intervention vs. comparison)**		**Time effect (Post-test vs. pretest)**		**Intervention effect (Community x time)**	
		**Pretest**	**Post-test**						
		**Mean(SD)/%**	**Mean(SD)/%**	**Adjusted B/OR**	**P**	**Adjusted B/OR**	**P**	**Adjusted B/OR**	**P**
**Restaurant Customer Data (N=721)**									
Satisfaction with fruit/vegetable choices, Mean (SD)	Intervention	2.41 (1.18)	2.80 (1.18)	-.18^a^	.589	**30** ^**a**^	**.004**	-.01^a^	.975
	Comparison	2.61 (1.11)	2.96 (1.08)						
Satisfaction with low-calorie choices, Mean (SD)	Intervention	1.76 (1.25)	2.34 (1.23)	-.077^a^	.726	**.42** ^**a**^	**.022**	.07^a^	.684
	Comparison	1.88 (1.27)	2.36 (1.14)						
Did you order any food promoted by materials/signs, *%*	Intervention	30.9	37.0	.83^b^	.717	**.52** ^**b**^	**.045**	2.23^b^	.094
	Comparison	42.7	30.8						
How healthy was last meal, *Mean (SD)*	Intervention	2.08 (1.30)	2.16 (1.41)	.06^a^	.849	.13^a^	.322	-.20^a^	.189
	Comparison	2.00 (1.35)	2.23 (1.35)						
**Store Customer Data (N=601)**									
Satisfaction with fruit/vegetable choices available at store, *Mean (SD)*	Intervention	3.01 (.91)	3.23 (.81)	**-.20** ^**a**^	**.000**	-.09^a^	.663	.35^a^	.147
	Comparison	3.19 (.78)	3.10 (.93)						
Satisfaction with promotion of fruit and vegetable choices, Mean (SD)	Intervention	2052 (1.05)	2.74 (1.00)	**-.09** ^**a**^	**.016**	-.10^a^	.832	.36^a^	.458
	Comparison	2.57 (1.07)	2.48 (1.18)						
Satisfaction with selection of low-calorie choices available at store, *Mean (SD)*	Intervention	2.43 (1.07)	2.58 (1.03)	**-.18** ^**a**^	**.001**	-.12^a^	.633	.28^a^	.353
	Comparison	2.57 (1.02)	2.45 (1.10)						
Satisfaction with promotion of low-calorie products, *Mean (SD)*	Intervention	2.10 (1.13)	2.25 (1.09)	.01^a^	.499	-.07^a^	.842	.26^a^	.474
	Comparison	2.04 (1.11)	1.96 (1.17)						
Did you purchase any food promoted by materials/signs, *%*	Intervention	28.6	27.1	**.73** ^**b**^	**.000**	1.39^b^	.157	.64^b^	.299
	Comparison	34.8	44.7						
How healthy was overall food purchase, *Mean (SD)*	Intervention	2.16 (1.26)	2.56 (1.05)	**-.42** ^**a**^	**.000**	.01^a^	.945	**.35** ^**a**^	**.022**
	Comparison	2.54 (1.07)	2.58 (1.19)						

^a^Adjusted coefficients (B) and p values are based on multiple linear regression models with cluster option (cluster variable = outlet where data came from). For restaurant data, models were adjusted for age, gender, education, residency, meal (lunch/dinner), day (weekend/weekday), and celebrating (yes/no). For store data, models were adjusted for age, gender, education, residency, day (weekend/weekday).

^b^Adjusted odds rations (AOR) and p values are based on logistic regression models with cluster option (cluster variable = outlet where data came from). For restaurant data, models were adjusted for age, gender, education, residency, meal (lunch/dinner), day (weekend/weekday), and celebrating (yes/no). For store data, models were adjusted for age, gender, education, residency, day (weekend/weekday).

Note. Significant interaction of the community and time effect would indicate changes overtime in intervention community compared to the comparison community. A significant interaction effect would suggest the campaign significantly impacted the outcome in the intervention compared to the comparison community.

Bold font indicates B regression coefficients or odds ratios statistically significant at p<=0.05.

In stores, satisfaction with the availability and promotion of fruit/vegetable and low-calorie choices improved slightly in the intervention community and worsened in the comparison community, but after adjusting for covariates these differences were not statistically significant, suggesting no intervention effects on these variables. Likewise, no effects were observed for the likelihood of ordering foods promoted as healthy in the store. We observed a greater increase in the perceived healthiness of the food purchased in the intervention community compared to the comparison community. Results from the adjusted regression model suggested a significant effect of the campaign on this variable (B=0.35, p=0.022; Table 4).

## Discussion

This study used the RE-AIM framework to evaluate the impact of WES, a pilot intervention to improve the nutrition environment and promote healthy eating in restaurants and supermarkets in a rural Midwest community. The results indicate high levels of outlet participation, with 78% and 66% of restaurants and supermarkets invited to participate agreeing to implement the intervention. The successful level of outlet recruitment can be attributed to an in-depth formative assessment and planning process. The coalition partners had strong relationships with community partners and restaurant and store operators. These relationships and knowledge helped to identify “early adopters” that might be willing to participate in the intervention. A recruitment process involving three interviews with potential participants and a menu of intervention activities to choose from allowed for the tailoring of WES strategies to the needs and preferences of each outlet. Similar incremental approaches have been successful in previous healthy eating interventions in food stores [28].

WES demonstrated moderate to high levels of implementation and maintenance. Consistent with previous interventions in restaurants [29-32] and food stores [13,33,34], WES participating outlets successfully selected and implemented a high number of activities and most outlets maintained many WES activities 10-months after launching of the intervention. Whereas other studies allowed outlets substantial flexibility in implementing strategies, [35-41] our approach emphasized and formalized (through a signed agreement with the managers) which activities the outlet would try to implement to cover the three WES strategies (promotion, availability, and point of purchase [POP]). By requiring outlets to commit to certain types of broad strategies (e.g., promotion, availability, labeling) while offering flexibility in how these were implemented (e.g., signs on the counter, table, or staff pins), our project ensured consistency across outlets. It’s likely this flexibility improved the implementation and maintenance of activities, and, at the same time, increased consistency in the use of the three broad strategies across outlets.

Despite continued WES participation, there was a slight decline at post-test in the average number of activities in place in participating restaurants and supermarkets. Other restaurant and food store interventions have reported similar challenges to ensure sustainability of the program. [40,41]. Some activities seem easier to sustain (e.g., window cling, healthy meal availability), while other activities (e.g., promotion by wait staff, deli sample tasting stations) may be more difficult to sustain without continued support from dedicated NPA coalition staff. In our study, the slight decline in activity implementation may be attributable to a lower level of support from the NPA coalition after the research funding ended. To improve sustainability, future efforts should attempt to partner with diverse stakeholders such as local hospitals, health departments, and businesses, build community capacity, ensure adequate staffing, and strategically plan for long-term sustainability [42].

WES achieved broad customer awareness. The variety and number of materials in restaurants and stores along with popular newspaper promotions by a local celebrity likely helped raise customer awareness of the intervention. Overall, by the end of the 10-month evaluation period over half of customers at participating restaurants and stores had heard of WES and an even larger percentage recognized the WES logo. Previous restaurant interventions have reported reach levels (i.e. awareness among restaurant customers) ranging from 20 to 100% [15,39,43-48]. The seven participating restaurants and two stores represented one-third of all restaurants and one-half of food stores in the intervention community. This would suggest at least a moderate level of exposure among community residents. Future studies should expand evaluation methods to assess the level of reach at the community level (e.g., using communitywide household or phone surveys).

This pilot study was not designed to test effectiveness, but to demonstrate feasibility and acceptability and inform a future effectiveness community trial. Still, our pilot data suggest WES was associated with an improvement in nutrition environment scores for restaurants (almost 80% increase). However, no changes in the store nutrition environment and only limited effects on customer food purchases and perceptions were observed. The results regarding improvements in the nutrition environment of restaurants participating in WES are promising. Restaurant environment scores improved partly due to signage, identification of healthier foods, and promotion of healthier foods, which are measured by the NEMS-Restaurant survey. The lack of changes in store nutrition environment scores could reflect the limited number of food stores and/or a lack of fit between the aspects of the environment measured by the NEMS-Store (NEMS-S) survey and the WES strategies implemented in these outlets. NEMS-S focuses on availability, quality, and price of healthy options. Large supermarkets, such those participating in WES, tend to score high in quality, availability, and price compared to small food stores. For small food stores, these three dimensions of the nutrition environment represent important intervention targets [49]. However, interventions implemented in large food stores often aim to label and promote available healthy choices to influence customer purchases and cooking practices [50-53]. WES activities in stores included distribution of healthy recipes, signage promoting produce, and in-store displays with healthy deli samples. These strategies are not reflected in NEMS-S. Consequently, this tool may not have captured changes that actually took place in the intervention store environments. Direct observation of WES activities indicated that participating stores made changes to modify their environments in the expected direction (e.g., displayed point-of-purchase signs for fruits/vegetables). In a future effectiveness trial, a larger number of food stores should be included and environment audit tools focused on marketing and promotional practices, rather than availability, quality, and price, should be used, in order to better test the potential effectiveness of this intervention approach.

Our pilot results show only minimal changes in customer attitudes and behaviors. Customer’s satisfaction with fruits and vegetables and low-calorie choices improved similarly in both the intervention and comparison community. These changes may reflect a general trend in local restaurants to increase F&V and low-calorie choices. The formative research that informed our intervention revealed restaurant owners/operators perceived a slow trend in this direction in response to increasing customer demand for these food options (Assessing the Nutrition Environment in Wisconsin Communities, unpublished data). Alternatively, the changes may reflect seasonal variations in restaurant offerings. The campaign was associated with a marginally significant increase in the likelihood of purchasing foods promoted as healthy in intervention restaurants. Ratings of the helpfulness of WES materials by restaurant customers were disappointingly low (an average of 0.9 on a 0 to 4 scale), perhaps reflecting than knowledge and availability of healthy options are insufficient to help restaurant customers make healthy choices. The limited impact suggests eating healthy is not a priority, and may be even antithetical to the notion of “eating out”, for many individuals. Interventions to change, not only the restaurant food environment, but also the culture around dining out may be required to more effectively promote healthy food choices in restaurants.

Customers in intervention stores showed significant, but small improvements in the reported healthiness of their purchases compared to customers in control stores. Lack of, or modest, effectiveness at the customer level has been found for other similar interventions in supermarkets [54-56] and restaurants [57-59]. The lack of clearer results could be attributable to limited statistical power to detect small effects. Community-based interventions in real-world conditions often report small size effects compared to highly controlled efficacy trials [17]. Environmental changes may take time and greater dosage to influence individuals. The 10-month intervention may have been too brief and/or of insufficient intensity to translate into individual level behavioral changes. Implementation of the intervention in a higher number of outlets in the intervention community could have resulted in greater impact on customer attitudes and choices. Complementary and targeted interventions to increase nutrition literacy, improve attitudes, and shift social norms regarding healthy foods may be necessary to influence the impact of restaurant-and food store-based interventions on customer behaviors and attitudes.

Overall findings from this study are consistent with previous literature that shows good evidence on the feasibility and acceptability, but mixed findings regarding effectiveness of community-based healthy eating interventions in restaurants and grocery stores [11-13]. Our study was one of few implemented in a rural community and among the first to show improvements in restaurant nutrition environment scores following implementation of the intervention. These promising findings call for additional effectiveness research to expand the evidence base regarding interventions that positively impact the nutrition environment and increase understanding of the association between the nutrition environment and food purchasing behaviors.

### Study limitations

This pilot study is subject to several limitations. The randomized community trial design, with only one community per condition allows for multiple validity threats. Selecting meaningful intervention and comparison communities is difficult in real world conditions. Despite our best efforts to select two comparable communities without major healthy eating activities, some healthy eating activities occurred in the comparison community during the intervention period, such as a community weight-loss program, worksite wellness programs, and local food system development. The driving distance between the two communities was almost 30 miles. The short distance and differences in size (6,000 versus 26,000 residents for the intervention and comparison communities, respectively) makes it likely for residents of the smaller city to travel to the larger one for meals and shopping. However, we believe this issue should not affect the validity of our results, given that (a) the samples were drawn from the restaurants and stores in each city, (b) we measured and adjusted for whether participants lived in the city or were visiting, and (c) survey questions were referred to their most recent experience dining or shopping in the outlet where they were recruited (versus experiences in other restaurants and/or communities).

With the exception of the Nutrition Environment Measurement Survey (NEMS), measurement instruments and procedures were developed ad hoc for this study. The reliability and validity of these measures will have to be evaluated to inform refinement of measures and procedures in a future larger study. Other limitations include a relatively short 10-month intervention time frame and lack of sales data. An effort was made to collect sales data quarterly from participating outlets. However, the variety of methods by which outlets tracked sales (e.g. electronically, paper slips, etc.) and our limited success in obtaining high-quality sales data dissuaded us from using these data to evaluate the effectiveness of this evaluation. Future studies should strive to include sales data and enhanced measures of food orders and purchases to better ascertain whether a similar health eating intervention changes food ordering and purchasing decisions. Despite these limitations, this study provides important pilot data to inform future research examining the impact of healthy eating interventions in restaurants and food stores in rural communities.

## Conclusions

Interventions to improve the nutrition environment in restaurants and supermarkets represent a promising approach to promote healthy eating behaviors, decrease the burden of obesity, and improve population health. Results from this study suggest that community-based nutrition interventions in local restaurants and supermarkets of a rural community can have a high level of reach and moderate levels of adoption, implementation, and maintenance. Our findings demonstrate these interventions are feasible and acceptable and could be effective at improving nutrition environments in restaurants. More evidence is needed to document the impact of this intervention approach on the food environment of supermarkets and their effectiveness to influence consumer and owner attitudes and purchasing behaviors.

### Consent

Written (outlet operators) and verbal (customer surveys) informed consent was obtained from the study participants for the publication of this report and any accompanying images.
